# Sleepiness and injury risk in emergency medical service workers in Taiwan

**DOI:** 10.1371/journal.pone.0229202

**Published:** 2020-02-24

**Authors:** Ming-Hung Lin, Yin-Chun Huang, Wei-Kung Chen, Jong-Yi Wang

**Affiliations:** 1 Department of Pharmacy and Master Program, Tajen University, Pingtung, Taiwan; 2 Department of Public Health, China Medical University, Taichung, Taiwan; 3 Nantou County Fire Bureau, Nantou, Taiwan; 4 Department of Emergency Medicine, China Medical University Hospital, Taichung, Taiwan; 5 Department of Health Services Administration, China Medical University, Taichung, Taiwan; Universitat de Valencia, SPAIN

## Abstract

**Background:**

Insufficient sleep is a common health and safety risk factor in high-impact workplaces where workers are required to take rotating shifts. However, studies on sleepiness-related risks and incidents, particularly among emergency medical services (EMS) workers are limited.

**Objective:**

This study sought to investigate the prevalence of sleepiness and related workplace incidents among EMS workers.

**Methods:**

This study utilized a cross-sectional survey design on a convenient sample of 500 EMS workers from 41 EMS squads across Taiwan. Data were collected using structured online questionnaires on workplace sleepiness and related safety incidents based on the Epworth Sleepiness Scale (ESS) and a modified 25-item EMS Safety Inventory respectively.

**Results:**

With a response rate of 79.8% (n = 399), 36.9% of the respondents were identified as having mild daytime sleepiness, while 39.2% of the respondents were identified as having excessive daytime sleepiness. Multivariate analysis indicated that not only was working on rotating shifts the main cause of the high ESS scores among EMS workers, but also that higher ESS scores increased their risk of sustaining a workplace injury. Furthermore, ill-at-work incidents were associated with an increased risk of workplace-related injuries.

**Conclusion:**

Overall, the findings indicated a correlation among working on rotation shifts, the prevalence of sleepiness, and a higher risk of workplace injury among EMS workers.

## Introduction

Fire departments across Taiwan, through their emergency medical services (EMS), are responsible for the provision of free around-the-clock ambulatory care services to patients who need an urgent medical response [1]. Since it was founded in 1995, the EMS system has witnessed a continued increase in demand for its services across the country. The workload for the EMS system increased from an estimated 678,989 calls in 2005 to 1,078,727 calls in 2014 [2]. With the increased demand for the EMS services, the prevalence of sleepiness is high among the EMS workers, which leads to work related injuries.

Sleepiness refers to a strong desire to fall asleep [3]. To show the seriousness of the sleepiness in the EMS department, it is worthy to put some statistics into perspective. According to Patterson [4], 50% of the EMS workers only had six hours of sleep for every 24 hours, while more than 50% workers reported experiencing poor quality sleep. EMS is a first aid squad that works on rotating shift schedules. Because EMS in Taiwan struggles with the lack of man-power, the use of a 48 hour-on and 24 hour-off shift rotation has been a concern. There are numerous factors that lead to the risk of workplace injury, among which the key factors includes age, health status, and sleepiness [5].

A Japanese study found that among 227 medical residents, 28.1% had excessive sleepiness. Furthermore, 45% of 12-hour-shift nurses indicated that they had a high level of sleepiness on the latest shift [6]. Sleepiness in health care providers has been a concern in patient care and safety [7–8]. A recent population-based study on EMS in Taiwan found that more than 70% of EMS providers had poor sleep [9]. Insufficient sleep and long work hours can be hazardous to the health of healthcare workers, and can cause health related incidents to those who receive care [10–11]. Sleepiness has been an important issue in research on traffic safety. Studies have found that traffic accidents are significantly related to sleepiness among drivers [12–15]. A study on 36,473 citizens in the general population also indicated that poor sleep relates to a 67% increase in the odds of traffic injuries [38]. A Canadian population-based study showed that women working rotating shifts are vulnerable with an elevated risk of injury at work [16].

Sleep deprivation can also affect physicians’ clinical performance and patient safety, particularly affecting resident physicians [17–20]. Night shift nurses with sleep deprivation are also more likely to make errors in patient care [21]. However, studies on work performance and job safety in EMS workers are relatively limited [22–23]. Equally, the health status of the workers in the EMS is an important factor that influences the performances of the personnel. In particular, workers with pre-existing health conditions are more likely to experience injuries. A New Zealand study indicated that people with pre-existing medical conditions have an odds ratio of 1.7 for an injury in the past 12 months [24]. Moreover, a survey conducted on Los Angeles taxi drivers showed that desirable health and less stress were associated with a large reduction in injury [25].

It has also been found that most EMS workers lack the information on how sleepiness affects their health and impacts the quality of the healthcare services that they provide to the patients [26]. Because of the lack of information regarding sleepiness and its potential impacts among the EMS workers, this topic is justified to be investigated as the findings will help improving the working conditions for the EMS workers. The cause of high prevalence of sleepiness among the EMS workers is attributed to the lack of sufficient personnel in the departments [27–29]. This study intended to investigate the sleepiness pattern and injury risk among EMS workers in Taiwan.

## Methods

### Study design and population

The target sample population for this study was the EMS workers of 12 fire departments located in northern, central, southern and eastern Taiwan. The first step in this study was to collect consents for survey from the fire stations. Each fire station consisted of two or three EMS squads based on the community population size, with four to seven EMS intermediates or EMS paramedics in each squad. The leaders of 41 EMS squads and 500 EMS workers responded to the survey invitation. Structured questionnaires were forwarded online to the subjects via a social media App. The respondents were able to click on the survey link from their smartphone or personal computer, and decide whether they would consent to participate in the survey. All data in the completed questionnaires were stored securely on a server. To encourage potential respondents to complete the survey on time, this study provided an 8GB Life-Star-printed USB drive as a gift to each respondent when the questionnaire was completed. The recruited participants were expected to complete the questionnaire based on their experiences over the past three months. The Research Ethics Committee of China Medical University & Hospital in Taiwan approved this study (CMUH103-REC1-113).

### Survey instrument

This study engaged a team of two EMS administrators and two senior emergency medical directors to evaluate the study questionnaire. The survey questionnaire included 13 items on socio-demographic status, five on workload, eight on the Epworth Sleepiness Scale (ESS) [30], and 25 on the Emergency Medical Service Safety Inventory (EMS-SI) [28]. The collected information included date of birth, height (cm), weight(kg), marital status, family size, EMT certification, job history and experiences with EMS squads, health status, lifestyle (smoking, drinking, and coffee and tea consumption, etc.), and workload on- and off- EMS missions such as shift rotation pattern and volume, call volume, and dispatches for fire disasters.

This study used the self-administered eight-item ESS to measure daytime sleepiness [30]. The ESS consists of eight specific scenarios, measured on a scale from 0 (not at all likely to fall asleep) to 3 (very likely to fall asleep), in order to identify how likely the respondents would feel sleepy in given scenarios. The sum of each respondent’s scores ranged from 0 to 24. The Chinese version of ESS was used in this study because it has been validated [31] as a standardized instrument to evaluate sleepiness for Mandarin-speakers [32].

To measure the EMS safety outcomes, this study adopted the Emergency Medical Service Safety Inventory (EMS-SI) developed with a modified Delphi-like iterative process by a panel of EMS experts. It was used by Patterson in the U.S. in 2011 [33]. Due to the different EMS circumstances and development between the U.S. and Taiwan, the 44-item inventory was modified and converted into 25 items. Three composites of safety outcomes were used, including 1) EMS worker injuries; 2) adverse events, and 3) compromised safety behavior. This study focused on the ESS status and injuries that had occurred to the EMS workers during the three months before the survey. A higher score indicates a poor safety outcome.

To evaluate the validity of the survey, this study designed the trap question “I made a patient with chest pain ambulate instead of using a stretcher”, which appeared twice in the questionnaire. The respondent would be defined as valid unless they had inconsistent answers for both.

### Data analysis

In order to measure the reliability and validity of the questionnaire, this study calculated the Cronbach’s alpha and Content Validity Index (CVI) of the ESS. A Cronbach’s alpha of 0.70 or is interpreted as a positive indicator of adequate reliability for the research instrument [34]and a CVI of 0.80 or above is considered a favorable outcome for instrument reliability and validity [35].

The mean, standard deviation (SD), and minimum and maximum scores were calculated to describe the overall findings of the ESS, injury, adverse events and compromised safety behavior. Frequencies and percentages were used to describe the three daytime sleepiness levels (normal, mild daytime sleepiness, and excessive daytime sleepiness) and the other demographic characteristics of respondents.

The primary objective of the inferential analysis was to evaluate the association between the respondents’ demographic characteristic, workload, ESS, and injury scores. This study used ANOVA to compare the means of the ESS and injury scores by demographic characteristics. Variables significant at the 0.05 level were included in the multivariable linear regression analysis to identify the effect of different sleepiness statuses and other variables in association with injury. Data analysis was performed using SPSS for Windows, Version 22.0 (SPSS Inc., Chicago, U.S.A.).

## Results

### Instrument reliability and validity

Cronbach’s alpha for ESS was 0.803 and the Content Validity Index score was 0.97 in this study, indicating that the research instrument has fair reliability and validity.

### Sample characteristics and overall findings

**Fig 1:**
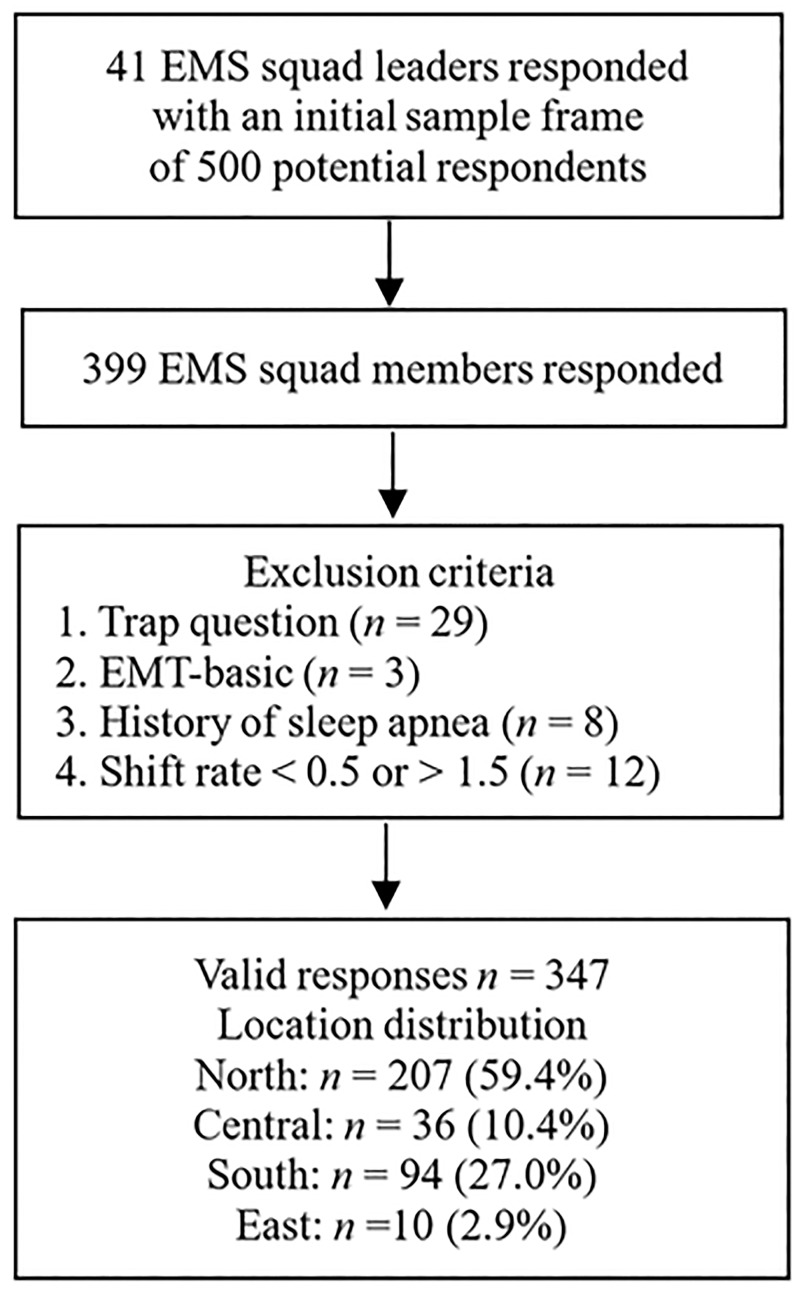
Flow chart of participant recruitment and work locations of the respondents.

A total of 500 EMT paramedics from 41 EMS squads affiliated with 12 fire departments located in northern, central, southern, and eastern Taiwan were contacted. Among them, 399 people responded to this survey (a 79.8% response rates) with consent. After excluding 29 respondents who did not answer the trap question consistently, there were 347 valid samples. Among the 29 questionnaires excluded, eight reported sleep apnea, three did not qualified for this survey, and 12 reported abnormal shift statuses (working either below 0.5 times or above 1.5 times the normal shift rate) (Fig 1).

The ESS scores reported by the 347 participants ranged between 0 and 24 with a mean of 10.6 (SD 4.2) (Table 1). The injury scores ranged from 0 to 2 with a mean of 1.0 (SD 0.6); the adverse event scores ranged from 0 to 10 with a mean of 2.4 (SD 2.1); and the mean of the safety compromised behavior scores ranged from 0 to 12 with a mean of 7.9 (SD 2.5). Table 2 shows that 83 participants (23.9%) have a normal sleep level (ESS <8), 128 (36.9%) have mild sleepiness (ESS 8~11) and 136 (39.2%) have excessive daytime sleepiness (ESS≧12).

**Table 1:** ESS, injury, adverse events, and compromised safety behavior scores of the study sample (*n* = 347).

Characteristic	Minimum	Maximum	Mean	SD
ESS score	0	24	10.6	4.2
Injury score	0	2	1.0	0.6
Adverse event score	0	10	2.4	2.1
Compromised safety behavior score	0	12	7.9	2.5

**Table 2:** Sleepiness level among the 347 respondents.

Characteristic	Number	Percent
Normal (ESS < 8)	83	23.9
Mild daytime sleepiness (ESS 8~11)	128	36.9
Excessive daytime sleepiness (ESS ≥12)	136	39.2
Total	347	100.0

### Factors associated with ESS and injury scores

Table 3 shows that 96.3% of the respondents are men, and the workers aged 26–30 years old are the major force. More than half of the respondents have less than five years of EMS experience.

**Table 3:** Mean Epworth Sleepiness Scale (ESS) and injury scores estimated by demographic factors, lifestyle, and workload (n = 347).

	Study sample		ESS			Injury score		
	*n* (%)		Mean	(SD)	*p*-value	Mean	(SD)	*p*-value
**Demographics**								
Gender								
	Female	13 (3.7)	10.23	3.68	*p* = 0.756	1.00	0.58	*p* = 0.972
	Male	334 (96.3)	10.60	4.24		0.99	0.62	
Age, years								
	20~24	31 (8.9)	9.48	3.65	*p* = 0.046 *	0.61	0.56	*p* = 0.001 **
	25~29	140 (40.3)	10.33	3.88		0.98	0.61	
	30~34	95 (27.4)	10.42	4.50		1.03	0.61	
	≥35	81 (23.3)	11.65	4.51		1.12	0.60	
BMI, Kg/M^2^								
	Normal (18.5~24)	135 (38.9)	10.39	3.96	*p* = 0.476	0.93	0.63	*p* = 0.140
	Overweight (> 24)	212 (61.1)	10.72	4.38		1.03	0.60	
Marital status								
	Single/divorced/widowed	162 (46.7)	10.40	3.87	*p* = 0.441	0.95	0.63	*p* = 0.215
	Married	185 (53.3)	10.75	4.50		1.03	0.60	
Number of children								
	0	194 (55.9)	10.43	3.96	*p* = 0.064	0.95	0.63	*p* = 0.293
	1	64 (18.4)	9.91	4.16		1.05	0.55	
	≥2	89 (25.6)	11.43	4.69		1.06	0.63	
Certification								
	EMT-intermediate	121 (34.9)	10.60	4.47	*p* = 0.960	0.79	0.60	*p*< 0.001 ***
	EMT-paramedic	226 (65.1)	10.58	4.08		1.10	0.59	
Years at fire experience								
	1~5	99 (28.5)	10.62	4.55	*p* = 0.392	0.92	0.60	*p* = 0.081
	6~10	134 (38.6)	10.24	3.75		0.96	0.63	
	> 1	114 (32.9)	10.97	4.43		1.10	0.60	
Years in EMS squad								
	1~2	89 (25.6)	10.46	4.31	*p* = 0.930	0.89	0.57	*p* = 0.066
	3~4	106 (30.5)	10.61	4.33		0.95	0.64	
	5~7	82 (23.6)	10.83	3.97		1.12	0.64	
	> 7	70 (20.2)	10.43	4.29		1.04	0.58	
Number of health problems								
	None	121 (34.9)	9.95	4.11	*p* = 0.005 **	0.76	0.67	*p*< 0.001 ***
	1	143 (41.2)	10.45	4.35		1.10	0.54	
	2	53 (15.3)	11.08	3.43		1.15	0.56	
	> 2	30 (8.6)	12.93	4.56		1.17	0.53	
Alcohol drink per week								
	0 days	233 (67.1)	10.67	4.48	*p* = 0.831	1.00	0.62	*p* = 0.909
	1	77 (22.2)	10.32	3.62		0.96	0.60	
	2	27 (7.8)	10.89	3.65		1.00	0.68	
	3	10 (2.9)	9.80	3.94		1.10	0.57	
Smoking								
	No	282 (81.3)	10.62	4.33	*p* = 0.764	0.99	0.63	*p* = 0.933
	Yes	65 (18.7)	10.45	3.74		1.00	0.56	
**Managing sleepiness**								
Coffee cup per day								
	0	209 (60.2)	10.15	4.11	*p*< 0.001 ***	0.97	0.63	*p* = 0.044*
	1	113 (32.6)	10.86	4.08		1.02	0.57	
	2	15 (4.3)	11.20	3.99		0.87	0.64	
	> 2	10 (2.9)	15.80	4.98		1.50	0.53	
Tea, cup per day								
	0	144 (41.5)	10.24	4.36	*p* = 0.165	0.99	0.60	*p* = 0.481
	1	122 (35.2)	10.55	3.87		0.98	0.61	
	2	48 (13.8)	10.73	3.99		0.96	0.65	
	> 2	33 (9.5)	12.06	4.94		1.15	0.62	
**Workload**								
Shift rotation style								
	24 on/24 off ^1^	211 (60.8)	10.58	4.30	*p* = 0.273	0.98	0.64	*p* = 0.644
	48 on/24 off with 3 extra days off ^2^	103 (29.7)	10.26	4.01		1.05	0.57	
	48 on/24 off with 2 extra days off ^3^	15 (4.3)	12.53	4.50		0.87	0.74	
	48 on/24 off with 1 extra day off ^4^	18 (5.2)	10.89	4.04		0.94	0.42	
Shift volume (based on a 25/75 percentile)								
	≤ 40	113 (32.6)	10.35	4.08	*p* = 0.310	0.96	0.60	*p* = 0.701
	41~45	157 (45.2)	10.44	4.21		1.01	0.63	
	> 45	77 (22.2)	11.23	4.41		1.03	0.61	
Call volume (based on a 25/75 percentile)								
	≤ 105	87 (25.1)	9.63	4.10	*p* = 0.045 *	0.95	0.66	*p* = 0.501
	106~210	176 (50.7)	10.82	4.33		0.98	0.60	
	> 210	84 (24.2)	11.08	3.99		1.06	0.59	
Dispatch assignment in fire disasters								
	Firefighting^5^	170 (49.0)	10.77	4.34	*p* = 0.430	1.04	0.65	*p* = 0.222
	EMS / fire assistance^6^	177 (51.0)	10.41	4.10		0.95	0.57	

* Indicates *p*< 0.05,

** indicates *p*<0.01,

*** indicates *p*< 0.001 via ANOVA.

^1^ Respondents take a 24-hour shift and have 24 hours off consecutively.

^2^ Respondents take a 48-hour shift and have 24 hours off with three extra days off per month.

^3^ Respondents take a 48-hour shift and have 24 hours off with two extra days off per month.

^4^ Respondents take a 48-hour shift and have 24 hours off with one extra day off per month.

^5^ Respondents are dispatched to suppress the fire with full personal fire protective equipment on the front line when a fire disaster occurs.

^6^ Respondents perform fire assistance or are on standby for emergency medical service provision when a fire disaster occurs.

The mean ESS scores between female and male EMS workers were similar. The mean scores rose with an increase in age and the extent of health problems, but was inversely related to the amount of coffee drinks consumed. All means were close to the 10 ESS level or greater. Workers aged 35 years and older had a mean ESS of 11.6, while those who drank three cups of coffee or more at work had a low mean ESS score overall. Specifically, EMS workers who do not drink coffee were estimated to be 1.5 times more likely to sustain a work-related injury, as compared with those that drink coffee. The ESS scores also increased with the number of EMS calls; those who had 210 calls had a mean of score 11.1.

Among the 347 respondents, 281 respondents (81.0%) reported one injury in the past three months. Table 3 shows that the mean injury scores also increased with age and the extent of health problems. The mean injury was nearly two-fold higher for the oldest workers than for the youngest workers (1.12 vs. 0.61). The EMT paramedics had a higher mean injury than the EMT intermediates (1.10 vs. 0.79, P<0.01). Multivariable regression analysis showed that compared to the normal respondents, the injury scores were 0.173 times higher for mild sleepiness (P<0.05) and 0.193 times higher for excessive sleepiness (P<0.05) (Table 4). The oldest group had a score that was 0.278 times higher than the youngest group. The injury scores for the EMT paramedics were 0.224 times higher than for the EMT intermediates. Workers with health problems and those who drink more coffee also had higher injury scores. As shown in Table 3, people with the highest ESS and injury scores drink more than two cups of coffee. However, the sample size of the respondents is also smaller than people who drink only one cup of coffee. The small sample size resulted in larger mean ESS and also injury scores (lower power).

**Table 4:** Factors associated with injury (n = 347).

		Dependent variable: injury score			
		Unstandardized beta coefficients	Std. error	standardized beta coefficients	Sig.
(Constant)		0.312	0.119		0.009**
Daytime sleepiness					
	Normal (ESS < 8)	—	—	—	—
	Mild sleepiness (ESS 8~11)	0.173	0.081	0.136	0.034*
	Excessive sleepiness (ESS ≥ 12)	0.193	0.081	0.154	0.017*
Age (years)					
	22–24	—	—	—	—
	25–29	0.216	0.118	0.173	0.070
	30–34	0.221	0.125	0.161	0.078
	≥35	0.278	0.130	0.192	0.033*
	EMT-intermediate	—	—	—	—
	EMT-paramedic	0.224	0.069	0.175	0.001**
Number of health problems					
	0	—	—	—	—
	1 sort	0.288	0.072	0.232	<0.001 ***
	2 sorts	0.376	0.097	0.221	<0.001 ***
	> 2 sorts	0.326	0.120	0.150	0.007**

* Indicates p < 0.05,

** indicates p <0.01,

*** indicates p < 0.001 via ANOVA

## Discussion

EMS workers are injured more often than other medical professionals at work [36]. Many occupational injuries incurred by EMS workers are associated with fatigue and sleepiness due to longer work hours. Studies have attempted to identify ways in which EMS workers can recover better between shifts [37]. This cross-sectional study demonstrated a significant correlation between daytime sleepiness and injury in EMS workers. The injury score increased with the daytime sleepiness level. Furthermore, as evidenced in Table 3, our study findings indicated that more coffee has an inverse correlation with injury scores. Sleepiness is a confounding factor that affects both caffeine consumption (independent factor) and injury score (dependent variable). Further data analysis showed that a higher adverse event score, and a higher compromised safety behavior score were also indicators of injury.

### Sleepiness

Our results showed that excessive daytime sleepiness is common among EMTs in Taiwan. While this study found a mean ESS score of 10.6 with a standard deviation of 4.2 from the questionnaire survey. An Australian study showed that normal adults have a mean ESS score of 4.6 with a standard deviation of 2.8 [38]. In this study, 39.2% of the EMS workers showed excessive daytime sleepiness with an ESS of≥ 12. This was much higher than the findings in other studies, which have shown rates of 8.6% for general citizens aged 15 years or older [39], 5.1% for college students [40], 11.9% for other types of factory workers [41], and 28% for police officers [42]. In our study, a higher ESS score was associated with older age, health problems, and a greater call volume. In contrast, the sleepiness score was not significantly associated with shift volume. A possible explanation of this finding was that a regular EMT shift in Taiwan is a 24-hour shift, and nearly 75% of the respondents had at least 41 shifts in the past three months. However, the frequency of calls could deprive the subjects of regular sleep and cause them to develop sleepiness at duty.

### Injury

The finding of this study shows that sleepiness is a precipitating factor for injuries among the EMS workers. This finding is in agreement with the existing literature which demonstrates that sleepiness affects the performance levels of workers negatively as it impairs alertness [43–46]. The similarity between this finding and previous literature is attributable to the observation that when one’s body is deprived of sufficient sleep, a compensatory mechanism occurs in which there is a need for the person to sleep first before the body gets back to its normal rhythm [47]. Our further data analysis showed that the respondents had a much greater injury rate (81.0%) and adverse event rate (81.6%). These findings urge for the overall EMS safety performance in Taiwan. The multivariable analysis also demonstrated that a higher daytime sleepiness level was related to poor EMS safety outcomes. Thus, this study demonstrated that sleepiness is a key risk factor concerning safety issues for EMS workers.

### Health status, workload, managing sleepiness, and safety

In addition to sleepiness, this study also showed that health status and workers’ skill levels were also associated with injury. Both the ESS and injury scores increased with the extent of health problems, suggesting that EMS workers with illness are at a higher risk of having sleepiness and encountering injury at work. This finding is also supported by the current literature, which shows that pre-existing health conditions increased the likelihood of injuries among workers in different occupations [48–50]. The consistency between the current findings and the existing literature is evidenced by the observation that the presence of pre-existing health conditions impairs the physiological functions of the body and in the process interferes with aspects such as concentration and activity levels [51]. For example, a sick worker is more likely to experience sleepiness than a worker without a pre-existing health issue.

Consuming caffeine has been recommended to maintain alertness and fight sleepiness [52–54]. This study found statistically significant relationships among sleepiness, caffeine consumption, and safety outcome. Although it has been suggested that caffeine be consumed at the beginning of a shift and should be avoided at least three hours before any planned sleep [51], there exist many variations in the unique 24-hour shift setting. Further research is needed to study the management of sleepiness in 24-hour shift EMS providers.

In healthcare settings, a higher workload is associated with poorer safety outcomes [50–52]. Interestingly, this study failed to find a significant relationship between workload and injury, despite the heavy workload of the participants. A possible explanation is that although researchers have attempted to develop a tool to assess sleep, fatigue, and alertness among EMS workers [53], a golden rule for measuring EMS workload has yet to be defined.

In this study, EMS paramedics had a higher injury rate than EMS intermediates, although the sleepiness levels were similar in these two groups. EMS workers are responsible for providing emergent care to patients at the scene and for transporting them via ambulance to an emergency department. While emergency care requires high effort and alertness in patient care, injuries may occur because potential hazards may exist at the incident scene. EMS workloads vary; therefore there were many factors that could not be measured in this study, including the distribution of dispatch timing, the duration of a single call, the severity of the patients’ conditions, etc. In addition, the relationship between an extended work schedule and safety outcome has yet to be clearly identified^54^. However, a relationship between call volume and sleepiness was identified in this study, in that a higher personal call volume was related to a higher level of sleepiness. Sleepiness may be a mediator between workload and injury. The quality of the emergency care delivered between EMS workers and paramedics is different. As paramedics provide more aggressive treatment than EMS workers, it is reasonable to hypothesize that sleepiness could have a greater effect on paramedics than on EMS workers. Additional research is needed to identify the relationships between workload, sleepiness, and safety outcome.

### Limitations

Previous studies have shown some limitations in using the EMS-SI in a survey, such as recall bias and a tendency by respondents to under-report [17]. There were some other limitations in the present study. First, the generalization of the findings is limited, as this study used a convenience sampling design. The lack of an EMS-population-based study in Taiwan is a weakness, and this study did not properly compare the respondent demographics with those of other studies. However, this study provided information that could be used as a reference in future research. Second, this study adopted the EMS-SI instrument and assessed safety using continuous scores. Higher scores indicated a poor safety outcome with more reported safety-related items, however, limited information was available on the frequency and severity of each safety issue. The sample size of people taking more than two cups of coffee day was 10 and the implication of this small sample size for the study was that it resulted in larger mean ESS and also high injury scores (lower power). This was a limitation as it was a source of potential misinterpretation for the findings. Since caffeine is taken to control sleepiness, the interpretation could be misleading as it appears to demonstrate that increasing consumption of caffeine increases sleepiness and related injuries.

## Conclusion

In this study, 39.2% of the respondents experienced excessive daytime sleepiness. A correlation between sleepiness and injury was identified for EMS workers in Taiwan. Respondents with higher sleepiness levels reported poorer safety outcomes. In addition to sleepiness, EMS workers’ workload and health status also affected EMS safety. Those who had greater call volumes experienced higher levels of daytime sleepiness. Call volume is an important factor related to their sleep and alertness. These findings may serve as reference for future policy making on EMS safety.
